# Abdominal compartment syndrome after endoscopic combined intrarenal surgery

**DOI:** 10.1002/iju5.12537

**Published:** 2022-09-16

**Authors:** Shuhei Okada, Takuro Saito, Yasushi Ichimura, Masahiro Iinuma

**Affiliations:** ^1^ Department of Urology Mito Medical Center Higashiibaraki‐gun Japan

**Keywords:** abdominal compartment syndrome, endoscopic combined intrarenal surgery, hydroperitoneum, percutaneous nephrolithotripsy, staghorn calculus

## Abstract

**Introduction:**

We report a case of abdominal compartment syndrome due to hydroperitoneum after endoscopic combined intrarenal surgery.

**Case presentation:**

A 56‐year‐old woman with a left staghorn calculus underwent endoscopic combined intrarenal surgery as a two‐staged procedure and developed a distended abdomen, cyanosis of both legs, and hypotension immediately after the second operation. A computed tomography scan showed hydroperitoneum. We performed urgent laparotomy and evacuated approximately 2 L of nearly transparent fluid. No peritoneal injury was detected. Postoperatively, she required intensive care for shocked liver and acute kidney injury.

**Conclusion:**

Hydroperitoneum after endoscopic combined intrarenal surgery is a rare complication and may lead to abdominal compartment syndrome or a condition where intra‐abdominal pressure exceeds 20 mmHg, causing impaired organ perfusion. Delayed drainage can be fatal.

**Keynote message**

Hydroperitoneum is a rare complication of endoscopic combined intrarenal surgery. Immediate drainage is desirable.

**Abbreviations & Acronyms**
ACM‐IGP: air‐capsule‐based measurement of intra‐gastric pressure
ACS: abdominal compartment syndrome
CT: computed tomography
ECIRS: endoscopic combined intrarenal surgery
PNL: percutaneous nephrolithotripsy

## Introduction

ECIRS or PNL is widely performed to treat staghorn calculus. We report a case of hydroperitoneum, or leakage of irrigation fluid into the peritoneal cavity, after ECIRS. In this case, hydroperitoneum resulted in ACS, or a condition in which intra‐abdominal pressure exceeds 20 mmHg resulting in impaired organ perfusion.

## Case presentation

**Fig. 1:**
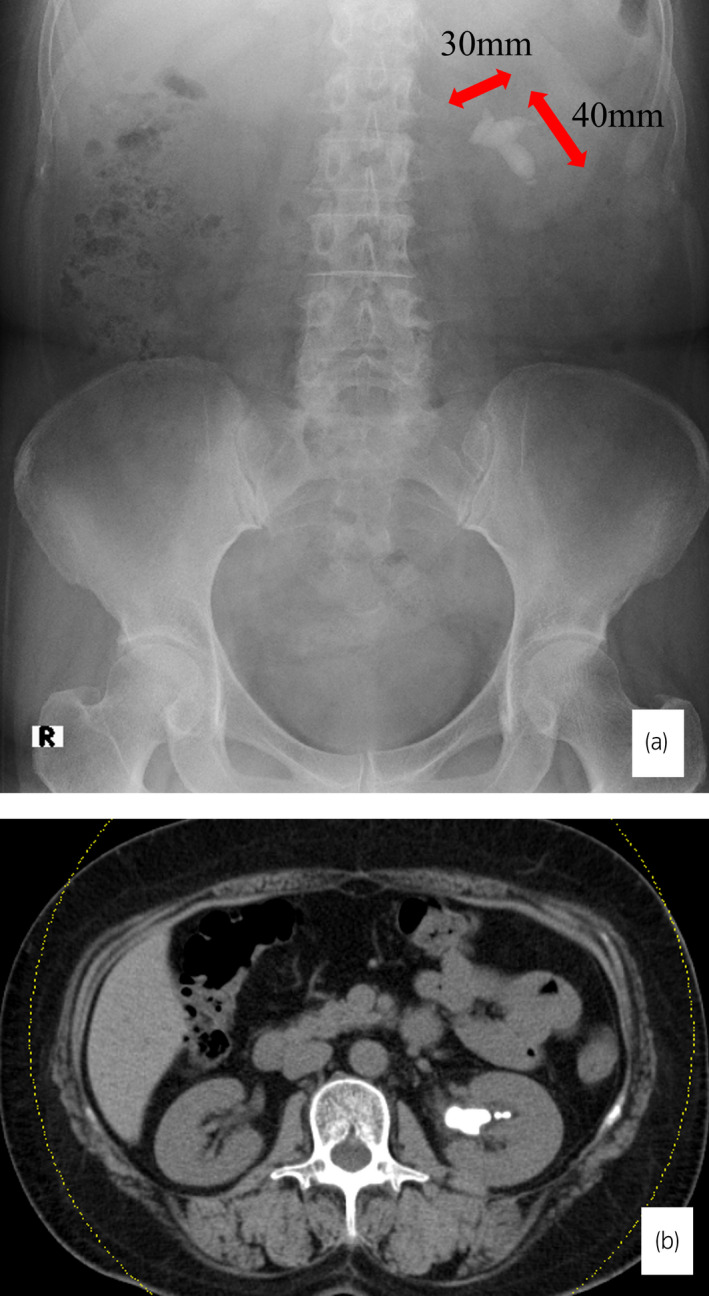
(a) A left staghorn calculus measuring 40 × 30 mm was observed. (b) Preoperative CT showed no anatomical risk factor of peritoneal puncture.

A 56‐year‐old woman was referred to our hospital because of asymptomatic gross hematuria. An abdominal radiograph revealed a left staghorn calculus measuring 40 × 30 mm (Fig. 1a). It involved the renal pelvis, middle, and inferior caliceal system. Left hydronephrosis was not observed. Contrast‐enhanced CT showed no anatomical risk factor for peritoneal puncture, such as anatomical abnormalities of the descending colon (Fig. 1b). ECIRS was performed as a two‐stage procedure in our department. The first‐stage surgery was conducted under general anesthesia on September 3, 2021.

The patient was placed in the modified Valdivia position. A 0.035‐mm guidewire was placed as a safety guidewire in the ureteral lumen (PIOLAX Tornado® [Piolax Medical Devices Inc., Yokohama, Japan]). We inserted a 12/14‐Fr 35‐cm ureteral access sheath (BARD Proxis® [Bard, New Providence, NJ, USA]).

We subsequently punctured the middle posterior calyx under ultrasound guidance. A 0.035‐mm guidewire (Boston Scientific Sensor® [Boston Scientific, Marlborough, MA, USA]) was inserted through the puncture needle, received by the flexible ureteroscope, and exited through the urethra. We dilated this percutaneous tract to 24 Fr using a balloon (Boston Scientific NephroMax®).

The antegrade‐side surgeon used a 22‐Fr nephroscope and Swiss LithoClast® (EMS, Nyon, Switzerland). The retrograde‐side surgeon used a 9.5‐Fr flexible ureteroscope (Boston Scientific LithoVue®) and a Holmium laser system (Lumenis Pulse 120H® and SlimLine SIS® 200 μm [Boston Scientific]). Irrigation fluid was located approximately 50 cm above the patient.

Although lever operation was not forceful, the flexible ureteroscope suddenly broke when operated at a downward angle, making a retrograde approach to the inferior calyx challenging. Thus, the first‐stage operation was terminated, and several stones remained in the inferior renal calyx. A 6‐Fr, 24‐cm ureteral stent (Boston Scientific Polaris Loop®) and a 24‐Fr nephrostomy tube were inserted. The operation time was 107 min, with an uneventful postoperative course.

We conducted the second‐stage surgery under general anesthesia on September 10, 2021. We achieved stone‐free status with an operation time of 114 min.

The patient was placed in the modified Valdivia position. The antegrade‐side surgeon placed a 24‐Fr sheath as the percutaneous tract (Boston Scientific Amplatz Type Renal Dilators®). The retrograde‐side surgeon inserted a 12/14‐Fr, 35‐cm ureteral access sheath (BARD Proxis®). Irrigation fluid was located approximately 50 cm above the patient. It was challenging for the antegrade‐side surgeon to approach the residual stone in the inferior renal calyx due to the renal column. To avoid injury to the kidney, the nephroscope was moved as little as possible. Therefore, we fragmented the residual stone using the ureteroscope and the fragments were removed via the percutaneous tract (pass the ball technique).

**Fig. 2:**
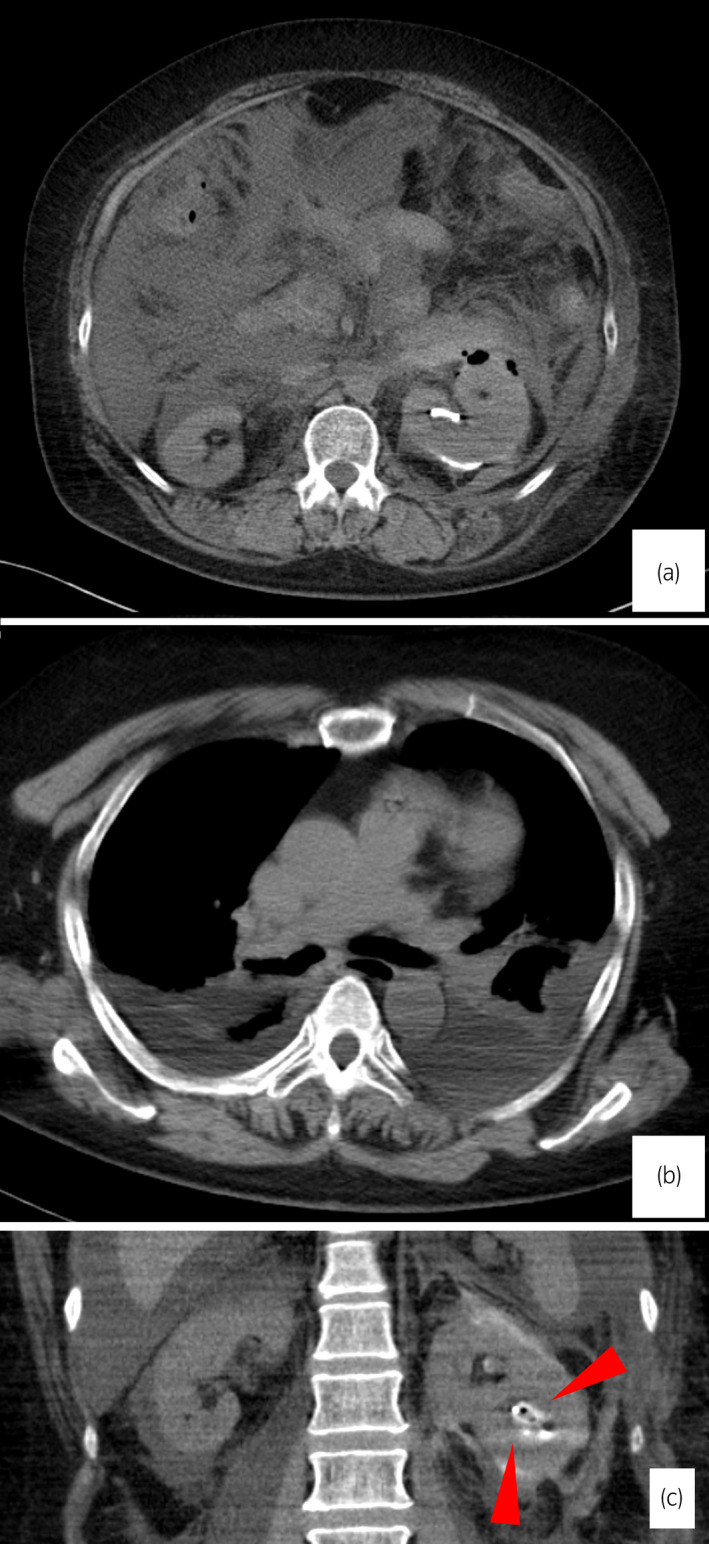
(a) A large amount of intraperitoneal fluid was observed despite little fluid accumulation in the retroperitoneal cavity. (b) Bilateral pleural effusion was observed. (c) Arrows show the nephrostomy tube tip located at the middle renal calyx.

Intraoperative bleeding was minimal, and renal pelvic or ureteral injury was not detected. Hemodynamic parameters remained stable, and airway pressure did not increase intraoperatively. However, when the drapes were removed, the patient's abdomen was markedly distended, and cyanosis in both legs was observed. Heart rate increased to 100 beats/min, and systolic blood pressure dropped to 70 mmHg. Diagnosis was difficult using only bed‐side ultrasonography; hence, a CT scan was performed (Fig. 2). Imaging revealed a large volume of intraperitoneal fluid and bilateral pleural effusion.

We performed an urgent laparotomy. Approximately 2 L of nearly transparent fluid was drained, but no peritoneal injury was detected.

It was difficult to inflate the bladder when placing a ureteral stent at the end of the second surgery. Therefore, intra‐vesical pressure, which reflects intra‐abdominal pressure, was inferred as more than 50 cmH_2_O or 36.8 mmHg. Thus, the case was diagnosed as ACS.

Postoperatively, the patient required intensive care for shocked liver and acute kidney injury. She was extubated after 9 days. The nephrostomy tube and the ureteral stent were removed on the 26th and 39th postoperative days, respectively. She was discharged on the 43rd day after the second‐stage operation.

## Discussion

We encountered a case of ACS due to hydroperitoneum after ECIRS. ACS is characterized by intra‐abdominal pressures exceeding 20 mmHg and impaired organ perfusion.^1^ It is a rare complication as, to our knowledge, only nine cases have been previously reported (Table 1).^2^, ^3^, ^4^, ^5^, ^6^, ^7^, ^8^

**Table 1:** Clinical features of the reported cases of hydroperitoneum after endoscopic combined intrarenal surgery

First author	Year	Laterality	Position	First sign	Treatment
Ghai	2003	Left	Prone position	Increased airway pressure	Percutaneous drainage
Etemadian	2010	Left	Prone position	Distended abdomen	Percutaneous drainage
Ozer	2012	Left	Prone position	Tachycardia	Percutaneous drainage
Tao No.1	2016	Right	Prone position	Increased airway pressure	Percutaneous drainage
Tao No.2	2016	Left	Prone position	Increased airway pressure	Percutaneous drainage
Benincasa No.1	2016	Left	Prone position	Distended abdomen	Percutaneous drainage
Benincasa No.2	2016	Right	Modified Valdivia position	Distended abdomen	Percutaneous drainage
Sharma	2018	Right	Prone position	Increased airway pressure	Percutaneous drainage
I.A. khalil	2021	Right	Modified Valdivia position	Distended abdomen	Percutaneous drainage
Okada	2022	Left	Modified Valdivia position	Distended abdomen	Laparotomy

Two mechanisms may result in hydroperitoneum. First, mucosal tearing may cause intravascular absorption of irrigation fluid. Second, a technical error in percutaneous tract placement may cause iatrogenic peritoneal injury. Khalil *et al*. reported that risk factors for hydroperitoneum include an increased amount of irrigation fluid, operative time of more than 60–90 min, renal pelvis mucosal tear, and peritoneal scarring caused by previous ECIRS or PNL.^8^

In this case, a minor peritoneal injury could have been overlooked. However, fluid overload should not be ruled out. It was reported that irrigation fluid is absorbed in all candidates who undergo PNL.^9^ It is also consistent with the bilateral pleural effusion in the present case.

In terms of preventing ACS due to hydroperitoneum, it is crucial to consider which position is superior between the prone and modified Valdivia positions. Renal calyx puncture is easier in the prone position,^10^, ^11^ but it is difficult to observe the patient's abdomen intraoperatively.

According to previous reports, increased airway pressure, distended abdomen, or tachycardia, was an early sign of hydroperitoneum.^2^, ^3^, ^4^, ^5^, ^6^, ^7^, ^8^ However, these signs may suggest a condition that led to ACS.

Intravesical or intra‐gastric pressure measurement is recommended to diagnose ACS.^12^, ^13^ Intravesical pressure cannot be measured accurately during ECIRS. Thus, intra‐gastric pressure measurement, such as ACM‐IGP, may be beneficial.^14^, ^15^

During the second surgery, 22 L of irrigation fluid was infused, and the output was 10 L. However, irrigation fluid leaked outside the operative field, making these measurements inaccurate. Proper draping of the operative field may prevent such occurrences.

Percutaneous drainage was conducted for treatment in all previously reported cases.^2^, ^3^, ^4^, ^5^, ^6^, ^7^, ^8^ It can be performed quickly, but its safety is questionable unless the fluid collection is visible on ultrasonography. Laparotomy is superior in terms of efficient drainage and surgical repair of abdominal organ injury. However, the mortality rate reaches up to 50%, even after surgical decompression.^1^ Therefore, prevention and early detection are crucial.

## Conclusion

Hydroperitoneum is a rare complication of ECIRS. It might lead to ACS or a condition in which intra‐abdominal pressure exceeds 20 mmHg resulting in impaired organ perfusion. Delayed drainage could be fatal.

## Author contributions

**Shuhei Okada:** Conceptualization; data curation; investigation; project administration; writing – original draft. **Takuro Saito:** Conceptualization; supervision. **Yasushi Ichimura:** Conceptualization; supervision. **Masahiro Iinuma:** Conceptualization; supervision; writing – review and editing.

## Conflict of interest

The authors declare no conflict of interest.

## Approval of the research protocol by an Institutional Reviewer Board

Not applicable.

## Informed consent

Informed consent for publication was obtained from the patient.

## Registry and the Registration No. of the study/trial

Not applicable.
